# *Ficus carica* L. (Moraceae): Phytochemistry, Traditional Uses and Biological Activities

**DOI:** 10.1155/2013/974256

**Published:** 2013-09-16

**Authors:** Shukranul Mawa, Khairana Husain, Ibrahim Jantan

**Affiliations:** Drug and Herbal Research Centre, Faculty of Pharmacy, Universiti Kebangsaan Malaysia, Jalan Raja Muda Abdul Aziz, 50300 Kuala Lumpur, Malaysia

## Abstract

This paper describes the botanical features of *Ficus carica* L. (Moraceae), its wide variety of chemical constituents, its use in traditional medicine as remedies for many health problems, and its biological activities. The plant has been used traditionally to treat various ailments such as gastric problems, inflammation, and cancer. Phytochemical studies on the leaves and fruits of the plant have shown that they are rich in phenolics, organic acids, and volatile compounds. However, there is little information on the phytochemicals present in the stem and root. Reports on the biological activities of the plant are mainly on its crude extracts which have been proven to possess many biological activities. Some of the most interesting therapeutic effects include anticancer, hepatoprotective, hypoglycemic, hypolipidemic, and antimicrobial activities. Thus, studies related to identification of the bioactive compounds and correlating them to their biological activities are very useful for further research to explore the potential of *F. carica* as a source of therapeutic agents.

## 1. Introduction

*Ficus* (Moraceae) comprises one of the largest genera of angiosperms with more than 800 species of trees, shrubs, hemiepiphytes, climbers, and creepers in the tropics and subtropics worldwide [1]. This genus is an important genetic resource due to its high economic and nutritional values and also an important part of the biodiversity in the rainforest ecosystem. It is also a good source of food for fruit-eating animals in tropical areas [2]. The genus is divided into six subgenera based on preliminary morphology. The monoecious subgenus *Urostigma* is the largest with about 280 species all inclusive, and most of them display distinctive hemiepiphytic habits. *Ficus* includes 23 species of hemiepiphytes and lithophytes which produce aerial and creeping root systems [3].

*F. carica* L. is an important member of the genus *Ficus. *It is ordinarily deciduous and commonly referred to as “fig”. The common fig is a tree native to southwest Asia and the eastern Mediterranean, and it is one of the first plants that were cultivated by humans. The fig is an important harvest worldwide for its dry and fresh consumption. Its common edible part is the fruit which is fleshy, hollow, and receptacle [4]. The dried fruits of *F. carica* have been reported as an important source of vitamins, minerals, carbohydrates, sugars, organic acids, and phenolic compounds [5–7]. The fresh and dried figs also contain high amounts of fiber and polyphenols [8, 9]. Figs are an excellent source of phenolic compounds, such as proanthocyanidins, whereas red wine and tea, which are two good sources of phenolic compounds, contain phenols lower than those in fig [10]. Its fruit, root, and leaves are used in traditional medicine to treat various ailments such as gastrointestinal (colic, indigestion, loss of appetite, and diarrhea), respiratory (sore throats, coughs, and bronchial problems), and cardiovascular disorders and as anti-inflammatory and antispasmodic remedy [11, 12].

*F. carica* L. belongs to the order of Urticales and family of Moraceae with over 1400 species classified into about 40 genera [13]. A number of them are functionally female and produce only a seed-bearing fruit, whereas others are functionally male and produce only pollen and pollen-carrying wasp progeny [14–16]. The species of *F. carica* are shrubs or small trees and deciduous. Its roots are not adventitious, and the barks are grayish and slightly roughened. The leaves are stipulated and petiolated with obovate, nearly orbiculate or ovate leaf blade, palmately lobed, cordate base, undulate or irregularly dentate margin, acute to obtuse apex, and scabrous-pubescent surfaces [17]. 

*F. carica *has been cultivated for a long time in various places worldwide for its edible fruit. It is supposed to originate from Western Asia and spread to the Mediterranean by humans [18]. It is also an imperative world crop today. Turkey, Egypt, Morocco, Spain, Greece, California, Italy, Brazil, and other places with typically mild winters and hot dry summers are the major producers of edible figs [19]. Fruits can be eaten raw, dried, canned, or in other preserved forms [20].

*F. carica *possibly originated from the Middle East, which is one of the early cultivated fruit species [21] and currently is an important crop worldwide. Nowadays, the common fig still grows wild in the Mediterranean basin. Morphological data propose that the fig is gynodioecious, whereas from a functional standing point, the fig is considered dioecious with two tree morphs: Capri fig and edible fig. Habitual fig cultivation areas have significantly decreased, and genetic variability was reduced due to disappearance of many cultivars selected in the past. Actually almost all grown cultivars are the result of old selection and are maintained by cutting as a way of vegetative propagation [22]. 

 The fruit (syconium or fig) and reproduction systems of species in the genus *Ficus *are exclusive. It can only be pollinated by their associated agaonid wasps (Hymenoptera: Chalcoidea: Agaonide), and in turn the wasps can only lay eggs within their associated fruit. For successful pollination and reproduction of species of *F. carica* to occur, its associated pollinator wasp must be present. Conversely, for successful reproduction of agaonid wasps to occur, their associated species of *F. carica* must be present [15]. The pollinator wasp for *F. carica *is *Blastophaga psenes* (L.) [23]. 

## 2. Phytochemistry

**Figure 1:**
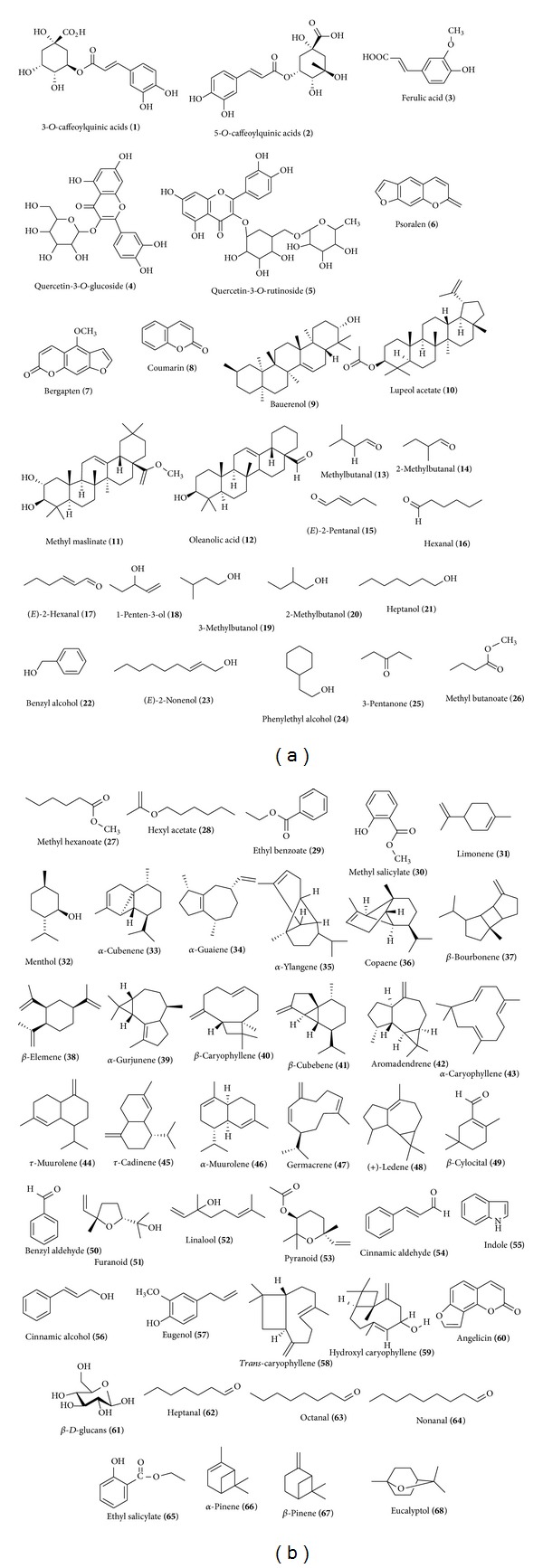
Compounds isolated from leaves, fruits, and barks of *F. carica *[24–28].

Phytochemical studies on *F. carica* revealed the presence of numerous bioactive compounds such as phenolic compounds, phytosterols, organic acids, anthocyanin composition, triterpenoids, coumarins, and volatile compounds such as hydrocarbons, aliphatic alcohols, and few other classes of secondary metabolites from different parts of *F. carica* (Figure 1). Most species of *F. carica* contain phenolic compounds, organic acids, and volatile compounds [24, 25].

Phenolic acids such as 3-*O*- and 5-*O*-caffeoylquinic acids, ferulic acid, quercetin-3-O-glucoside, quercetin-3-*O*-rutinoside, psoralen, bergapten, and organic acids (oxalic, citric, malic, quinic, shikimic, and fumaric acids) have been isolated from the water extract of the leaves of *F. carica* L. [24]. Coumarin has been isolated from the methanol extract of the leaves of *F. carica* L. by bioassay-guided isolation, and the isolated coumarin exhibited the strongest nematicidal activity against the nematodes *Bursaphelenchus xylophilus, Panagrellus redivivus, and Caenorhabditis elegans* within 72 hr [26]. Four triterpenoids, bauerenol, lupeol acetate, methyl maslinate, and oleanolic acid,have been isolated from the leaves of *F. carica* and showed irritant potential on mice ears [27].

The leaves of *F. carica* consist of various volatile compounds which are identified and distributed by distinct chemical classes, such as aldehydes: methyl-butanal, 2-methylbutanal, (*E*)-2-pentanal, hexanal, and (*E*)-2-hexanal, alcohols: 1-penten-3-ol, 3-methyl-1-butanol, 2-methylbutanol, heptanol, benzyl alcohol, (*E*)-2-nonen-1-ol, and phenylethyl alcohol, ketone: 3-pentanone, esters: methyl butanoate, methyl hexanoate, hexyl acetate, ethyl benzoate, and methyl salicylate, monoterpenes: limonene and menthol, sesquiterpenes: *α*-cubenene, *α*-guaiene, *α*-ylangene, copaene, *β*-bourbonene, *β*-elemene, *α*-gurjunene, *β*-caryophyllene, *β*-cubebene, aromadendrene, *α*-caryophyllene, *τ*-muurolene, *τ*-cadinene, *α*-muurolene, germacrene D, and (+)-ledene, norisoprenoid: *β*-cyclocitral, and miscellaneous compounds: psoralen [28].

Fifteen anthocyanin pigments were isolated from the fig fruit and bark of *F. carica.* Most of them contain cyanidin as aglycone and some pelargonidin derivatives [6]. Pentane extracts from the fig of *F. carica* contain numerous volatile compounds: benzyl aldehyde, benzyl alcohol, furanoid, linalool, pyranoid (*trans*), cinnamic aldehyde, indole, cinnamic alcohol, eugenol, and *trans*caryophyllenes sesquiterpene: germacrene D, hydroxyl caryophyllene, angelicin, and bergapten [25]. 

 Total and individual phenolic compounds, phenolic acid, chlorogenic acid, flavones, and flavonols, have been isolated from fresh and dried fig skins of *F. carica* and dried figs contained total higher amounts of phenolics than the pulp of fresh fruits, owing to the contribution of the dry skin. Quercetin rutinoside was the major individual phenolic [29] while microbial *β*-*D-*glucans has been isolated from Libyan figs of *F. carica *[30].

Phenolic acids; 3-*O*- and 5-*O*-caffeoylquinic acids, ferulic acid, quercetin-3-*O*-glucoside, quercetin-3-*O*-rutinoside, psoralen, and bergapten, and organic acids (oxalic, citric, malic, shikimic, and fumaric acids) were isolated from the pulps and peels of figs [24]. Phenolics, anthocyanins, fructose, glucose, and sucrose were identified from the fig of *F. carica* [31].

 Various volatile constituents of five Portuguese varieties of *F. carica* fruits (pulps and peels) have been isolated which include aldehydes: 3-methyl-butanal, 2-methyl-butanal, (*E*)-2-pentanal, hexanal, heptanal, octanal, and nonanal, alcohols: 1-penten-3-ol, 3-methylbutanol, benzyl alcohol, (*E*)-2-nonenol, and phenylethyl alcohol, ketone: 6-methyl-5-hepten-2-one, esters: methyl hexanoate, methyl salicylate, and ethyl salicylate, monoterpenes: limonene, menthol, *α*-pinene, *β*-pinene, linalool, eucalyptol, sesquiterpenes: *α*-cubenene, copaene, *β*-caryophyllene, *τ*-muurolene, *τ*-cadinene, and germacrene D, norisoprenoid: *β*-cyclocitral, and miscellaneous compounds: eugenol [28]. 

## 3. Traditional and Current Uses

*F. carica* has been traditionally used for its medicinal benefits as metabolic, cardiovascular, respiratory, antispasmodic, and anti-inflammatory remedy [11, 12]. It is commonly referred to as “Fig”. Leaves, fruits, and roots of *F. carica* are used in native medicinal system in different disorders such as gastrointestinal (colic, indigestion, loss of appetite, and diarrhea), respiratory (sore throats, cough, and bronchial problems), inflammatory, and cardiovascular disorders [32, 33]. Fruits of *F. carica* can be eaten fresh or dried or used as jam. Figs are used as an excellent source of minerals, vitamins, carbohydrates, and dietary fibre because it is fat and cholesterol free and contain high number of amino acids [6, 7, 34, 35]. It is also reported that figs have been conventionally used for their therapeutic benefits as laxative, cardiovascular, respiratory, antispasmodic, and anti-inflammatory remedies [36]. 

The fruit's juice of *F. carica *mixed with honey is used for haemorrhage. In Indian medicine, fruits are used as a mild laxative, expectorant, and diuretic [35]. It is used as aid in liver and spleen diseases. The dry fruit of *F. carica* is a supplement food for diabetics. It is commercialized in the market as sweet due to its high level of sugars [7]. Fruit paste is applied to swellings, tumours, and inflammation for relieving pain. Twenty-one traditional and current uses of *F. carica* including different ethnopharmacological reports. have been summarized in Table 1.

## 4. Biological Activities

### 4.1. Antioxidant Activity

*F. carica* contains many phenolic compounds that play many physiological roles in plants. Some of them are also favourable to human health, since they are able to act as an antioxidant by different ways: reducing agents, hydrogen donators, free radical scavengers, singlet oxygen quenchers, and so forth. Fig fruits of *F. carica *were studied with six commercial fig varieties with different colors (black, red, yellow, and green) for total polyphenols, total flavonoids, antioxidant capacity, and profile of anthocyanins. The antioxidant properties were determined by ferric reducing antioxidant method. Fruits contained the highest levels of polyphenols, flavonoids, and anthocyanins and exhibited the highest antioxidant capacity [31]. Fig fruits of *F. carica *were analyzed for total flavonoids, antioxidant capacity, and profile of anthocyanins [35]. Using RP-LC various concentrations of anthocyanins but similar profiles have been found in all varieties studied. Cyanidin was confirmed as the major aglycone in several studies [6, 34, 35]. NMR data confirmed that cyanidin-3-*O*-rutinoside (C3R) was the main anthocyanin in all fruits. Color appearance of the fig extract correlated well with total polyphenols, flavonoids, anthocyanins, and antioxidant capacity. C3R contributed 92% of the total antioxidant capacity of the anthocyanin fraction, and fruits contained highest levels of polyphenols; flavonoids and anthocyanins exhibited the highest antioxidant capacity [35].

### 4.2. Anticancer Activity

A mixture of 6-*O*-acyl-*β*-d-glucosyl-*β*-sitosterols has been isolated as an effective cytotoxic agent from fig (*F. carica*) latex that showed *in vitro* inhibitory effects on proliferation of various cancer cell lines [39, 41].

### 4.3. Hepatoprotective Activity

The petroleum ether extract from leaves of *F. carica* was evaluated for hepatoprotective activity on rats treated with 50 mg/kg of rifampicin orally, and significant reversal of biochemical, histological, and functional changes induced by rifampicin on rats indicated potential hepatoprotective activity [43]. 

### 4.4. Hypoglycamic Activity

The leaf extract induced a significant hypoglycamic effect in oral or intraperitoneal administration in streptozotocin-diabetic rats. Weight loss was prevented in treated diabetic rats, and plasma insulin levels considerably altered the survival index. Results indicated that the aqueous extract of *F. carica *has an obvious hypoglycemic activity [44].

### 4.5. Hypolipidemic Activity

The leaf extract of *F. carica* could be a beneficial supplement to modulate TG and TC secretion in poultry liver [45]. Eight-weeks-old rooster's liver with high abdominal fat was extracted, sliced, and cultured with increasing concentrations of leaf extract, insulin, and both of them. While insulin extensively increased TG secretion (0.190 ± 0.013 mmol/L), TG content (0.523 ± 0.093 mmol/L), and TC secretion (1.727 ± 0.412 mmol/L) beyond the basal level (*P* < 0.001) and when the leaf extract was added, the effects were drastically reduced to the basal level in a concentration-dependent manner (*P* < 0.001).

### 4.6. Antibacterial Activity and Anti-Fungal Activity

The methanol extract of *F. carica *(MICs, 0.156 to 5 mg/mL; MBCs, 0.313 to 5 mg/mL) showed a strong antibacterial activity against oral bacteria. The combination effects of methanol extract with ampicillin or gentamicin were synergistic against oral bacteria that showed that figs could act as a natural antibacterial agent [46]. Hexane, chloroform, ethyl acetate, and methanol extracts of *F. carica *latex were investigated for their antimicrobial proprieties *in vitro* against five bacterial species and seven strains of fungi using disc-diffusion method. The minimal inhibition concentration (MIC) of the methanol fraction showed a total inhibition against *Candida albicans *(100%) at a concentration of 500 *μ*g/mL and a negative effect against *Cryptococcus neoforman;* methanolic extract (75%) strongly inhibited *Microsporum canis* and ethyl acetate extract at a concentration of 750 *μ*g/mL [47].

### 4.7. Antipyretic Activity

The ethanol extract of *F. carica*, at doses of 100, 200, and 300 mg/kg, showed significant dose-dependent reduction in normal body temperature, and yeast provoked elevated temperature. The effect extended up to five hrs after drug administration while compared to that of standard antipyretic agent, paracetamol (150 mg/kg.b.wt., p.o.) [48].

### 4.8. Antituberculosis Activity

The 80% methanol extract from the leaves of *F. carica *has been screened against *Mycobacterium tuberculosis* H37Rv using a colorimetric microplate-based assay. The result exhibited anti-tuberculosis activity with MIC value of 1600 *µ*g/mL [40].

### 4.9. Irritant Potential

The methanol extract and isolated triterpenoids from the leaves of *F. carica* were tested for irritant activity. They exhibited irritant potential on mice ears, and calotropenyl acetate, methyl maslinate, and lupeol acetate were the most potent and importunate irritant is which were less than those of euphorbium and close to psoralen. Irritant potential was evaluated by open mouse ear assay [27].

### 4.10. Nematicidal Activity

Forty different plant species were screened for their nematicidal activity against the nematodes *Bursaphelenchus xylophilus, Panagrellus redivivus, and Caenorhabditis elegans*. The leaf extract of *F. carica* showed the strongest nematicidal activity as 74.3, 96.2, and 98.4% mortality, respectively, within 72 hrs [26].

### 4.11. Antispasmodic and Antiplatelet Activity

The aqueous-ethanolic extract of *F. carica* was investigated for antispasmodic effect on rabbit and antiplatelet effect using *ex-vivo* model of human platelets. *F. carica* was tested positive for alkaloids, flavonoids, coumarins, saponins, sterols, and terpenes, and when it was tested in isolated rabbit jejunum *F. carica* (0.1–3.0 mg/mL) produced relaxation of impulsive and low K^+^-(25 mM) induced contraction with insignificant effect on high K^+^ (80 mM) similar to that caused by cromakalim. Pretreatment of the tissue with glibenclamide caused rightward shift in the curves of low K^+^ but did not cause high potassium ion, while verapamil equally repressed the concentration of potassium ion at both concentrations. *F. carica* (0.6 and 0.12 mg/mL) repressed the adenosine-5-diphosphate and adrenaline-induced human platelet aggregation. That study exhibited spasmolytic activity in the ripe dried fruit of *F. carica* probably mediated through the activation of potassium ion ATP channels along with antiplatelet activity that provided sound pharmacological basis for its medicinal use in the gut motility and inflammatory disorders [37]. 

### 4.12. Anthelmintic

The anthelmintic activity of the latex of *F. carica* was investigated in NIH mice naturally infected with *Syphacia obvelata, Aspiculuris tetraptera,* and *Vampirolepis nana*. The latex was administered in doses of 3 mL/kg/day during three successive days, was effective in the removal of *S. obvelata* (41.7%), and did not produce significant elimination of *A. tetraptera *(2.6%) and *V. nana* (8.3%). High acute toxicity with hemorrhagic enteritis was observed; additional to a weak anthelmintic efficacy, was not recommended the use of this lattice in traditional medicine [49].

### 4.13. Antimutagenic

Antimutagenic activity of the plant extract of *F. carica* on environmental xenobiotics was investigated. The plant extract decreased the level of mutations induced by *N*-metil-*N*′-nitro-*N*-nitrosoguanidine (MNNG) in *Vicia faba* cells, chlorophyll mutations in *Arabidopsis thaliana,* and NAF induced mutability in rat marrow cells. The extract verified the ability to decrease the genotoxicity of environmental mutagens [50].

### 4.14. Anti-HSV

The water extract of the leaves of *F. carica *has been studied on anti-HSV effect and observed on Hep-2, BHK21, and PRK cells. The water extract possessed low toxicity and directly killing-virus effect on HSV. The MTC was 0.5 mg/mL, TDO was 15 mg/mL, and TI was 30.0 mg/mL [51].

### 4.15. Oxidative Stress

Oxidative stress was studied in rats divided into 4 groups: streptozotocin-induced diabetic rats (*n* = 10), diabetic rats that received a single dose of a basic fraction of *F. carica* extract (*n* = 14), diabetic rats that received a single dose of a chloroform fraction of the extract (*n* = 10), and normal rats (*n* = 10). Compared to normal animals, the diabetic animals exhibited extensively higher values for erythrocyte catalyze normalized to haemoglobin levels (1.5 ± 0.15 versus 0.96  ±  0.18 *µ*g/mg) and for plasma vitamin E (73.4  ±  43.9 versus 12.0 ± 1.6 mg/L), monounsaturated fatty acids (0.219 ± 0.118 versus 0.067 ± 0.014 mg/mL), polyunsaturated fatty acids (PUFA, 0.567 ± 0.293 versus 0.175 ± 0.040 mg/mL), saturated fatty acids (0.779 ± 0.262 versus 0.401 ± 0.055 mg/mL), and linoleic acid (0.202 ± 0.086 versus 0.106 ± 0.014 mg/mL). Both *F. carica* fractions showed that they normalize the values of the diabetic animal's fatty acids and plasma vitamin E values. They showed statistically significant differences as a function of diabetes with the vitamin E/C 18 : 2 ratio being normalized by the administration of the chloroform fraction (to 152.1 ± 80.3 *µ*g/mg) and the vitamin A/C 18 : 2 ratio being raised relative to the untreated diabetic rats by the administration of the basic fraction (91.9 ± 14.5 *µ*g/mg). That study confirmed that antioxidant status was affected in the diabetes syndrome, and *F. carica *extracts showed that they normalize it [52].

## 5. Conclusion

Many interesting biological activities of *F. carica* have been carried out, which can be further explored to make use of them as a healing method for the future. For example, the leaves have shown irritant activity; consequently they can be investigated against parasitic infection and ovicidal activity. The majority of the pharmacological studies which have been carried out on *F. carica* were conducted with uncharacterized crude extracts; it is difficult to produce the grades of these studies and identify the bioactive metabolites.

Phytochemical research carried out on *F. carica* has led to the isolation of few classes of plant metabolites. Most of the phytochemical works have been employed on leaves and fruits of *F. carica,* while there is little information on stem and root phenolic profiles. However the vast traditional uses and established pharmacological activities of *F. carica* point out that an enormous scope still exists for its phytochemical exploration using bioassay-guided isolation. The result of future research in the above mentioned areas will afford a persuasive support for the future clinical uses of *F. carica* in contemporary remedy.

## Figures and Tables

**Table 1:** Traditional and current uses of *F. carica. *

Uses	Part	Locality	Reference
Cough	Leaf	Malaysia	[37]
Colic treatment	Fruit, root, and leaf	unspecified	[32, 33]
Indigestion	Fruit, root, and leaf	unspecified	[32, 33]
Loss of appetite	Fruit, root, and leaf	unspecified	[32, 33]
Antidiarrheal	Fig	unspecified	[11, 12]
Metabolic	Fig	unspecified	[11, 12]
Cardiovascular	Fig	unspecified	[11, 12]
Respiratory	Fig	unspecified	[11, 12]
Antispasmodic	Fig	unspecified	[11, 12, 38]
Anti-inflammatory	Fig	unspecified	[11, 12]
Antiplatelet, inflammatory, and gut motility	Fig	Pakistan	[38]
Antioxidant	Fig	unspecified	[33]
Laxative	Fig	unspecified	[30]
Prevention of nutritional anaemia	Leaf	unspecified	[27]
Anthelmintic	Leaf	unspecified	[27]
Irritant potential	Leaf	unspecified	[27]
Nutritive diet	Fruit	Mediterranean countries	[31]
Various drug preparations	Fig fruit	unspecified	[39]
Tuberculosis	Leaf	Malaysia	[40]
Anticancer	Fig	unspecified	[41, 42]
Mild laxative, expectorant, and diuretic	Fruit	India	[40]
